# Influences of case-based inclusive education coursework and disability-related contact experiences on secondary preservice teachers' efficacy for inclusive practices: variable- and person-centered approaches

**DOI:** 10.3389/fpsyg.2026.1765852

**Published:** 2026-03-24

**Authors:** Weihao Xin, Boya Wang, Zhenzhen Zhang, Fuxin Lian

**Affiliations:** 1 Jing Hengyi School of Education, Hangzhou Normal University, Hangzhou, China; 2 Zhejiang Philosophy and Social Science Laboratory for Research in Early Development and Childcare, Hangzhou Normal University, Hangzhou, China; 3 Department of Special Education, Faculty of Education, East China Normal University, Shanghai, China

**Keywords:** case-based coursework, disability-related contact experiences, efficacy for inclusive practices, inclusive education, secondary preservice teachers

## Abstract

**Introduction:**

Little is known about how case-based inclusive education coursework and disability-related contact experiences, two increasingly emphasized components of contemporary teacher education, shape secondary preservice teachers' (SPTs') efficacy for inclusive practices.

**Methods:**

This study used variable-centered and person-centered approaches to examine the effects of a case-based inclusive education coursework intervention on Chinese secondary preservice teachers' efficacy for inclusive practices and to assess whether disability-related contact experiences shaped these outcomes. A sample of 483 SPTs completed the Teacher Efficacy for Inclusive Practices Scale before and after the coursework.

**Results:**

Variable-centered analyses showed that the case-based coursework significantly improved SPTs' efficacy for inclusive practices, with gains largely consistent across levels of disability-related contact experiences. Person-centered analyses identified three latent efficacy profiles—low, moderate, and high—which remained generally stable over time but also showed meaningful transitions. Disability-related contact experiences predicted the likelihood of these transitions.

**Discussion:**

These findings demonstrate the broad effectiveness of case-based coursework and illuminate differentiated developmental pathways in SPTs' efficacy for inclusive practices. The results provide important implications for strengthening preservice preparation for inclusive education.

## Introduction

Inclusive education has become a national priority in China, serving as a key pathway for advancing educational equity and promoting the social participation of students with disabilities (Zhang and Miao, 2022). According to the 2024 national education statistics (Ministry of Education of the People's Republic of China, 2024), among the 911,981 students enrolled in special education, 460,652 (50.51%) were studying in regular schools through full inclusion or in special classes attached to regular schools. Among them, 306,642 were in elementary schools, and 154,010 were in secondary schools. In response, national policies such as the *Regulations on the Education of Persons with Disabilities* (The State Council of the People's Republic of China, 2017) and the *14th Five-Year Special Education Improvement Action Plan* (General Office of The State Council of the People's Republic of China, 2021) emphasize the development of a systematic teacher preparation mechanism, including compulsory inclusive education coursework and strengthened professional support. These policy shifts highlight an urgent need to ensure that teachers entering the profession—especially those who will work with diverse learners—are adequately prepared for inclusive practices (Xin et al., 2025; Wang et al., 2018).

As the primary implementers of inclusive policies, teachers play a critical role in shaping the quality of inclusive practices (Xin et al., 2024a). However, inclusive teaching is particularly challenging in secondary schools, where subject-specialized instruction, exam-oriented pressures, and limited school-based support often restrict teachers' responsiveness to diverse learning needs (Fedha, 2025; Malak and Tasnuba, 2018). Many secondary teachers also lack knowledge of disabilities and practical experience with individualized instruction, both in preservice preparation and in subsequent professional development (Triviño-Amigo et al., 2022). These structural and professional challenges may undermine secondary teachers' efficacy for inclusive practices—an outcome strongly associated with effective inclusion (Wray et al., 2022).

Despite these concerns, existing research has focused predominantly on preschool and elementary teachers, whether preservice or in-service, providing limited insight into how secondary preservice teachers (SPTs) develop efficacy prior to entering the profession (Sharma, 2012; Wu and Luo, 2020; Zhao and Peng, 2021). In particular, little is known about how case-based inclusive education coursework and disability-related contact experiences—two increasingly emphasized components of contemporary teacher education—shape SPTs' efficacy for inclusive practices.

### Literature review

#### Teachers' efficacy for inclusive practices

Teacher efficacy, grounded in Bandura's (1977, 1997) work on self-efficacy, refers to teachers' beliefs about their capability to successfully perform instructional tasks and attain desired educational goals Within this broader construct, teacher efficacy for inclusive practices reflects teachers' judgments about their capacity to differentiate instruction, manage diverse classroom behaviors, and coordinate multiple supports in inclusive settings (Sharma et al., 2012). A growing body of research shows that these efficacy beliefs systematically shape teachers' instructional decision-making, professional motivation, and practice (Sharma and Nuttal, 2016). Teachers with stronger efficacy for inclusive practices tend to hold more positive attitudes toward students with disabilities (Long et al., 2025), take greater initiative in adapting curricula and designing individualized education plans (Woodcock and Hardy, 2025), and report higher professional accomplishment alongside lower burnout (Li, 2023). Given these associations, teacher education increasingly prioritizes cultivating efficacy for inclusive practices (Wang et al., 2018), with structured preparation in inclusive education identified as a primary mechanism for strengthening this efficacy (Wray et al., 2022). Because preservice preparation is the period in which efficacy beliefs consolidate and undergo critical calibration, it plays a pivotal role in shaping preservice teachers' (PTs) efficacy for inclusive practices (Xin et al., 2025).

#### Factors related to PTs' efficacy for inclusive practices

Prior research shows that PTs generally report moderate levels of efficacy for inclusive practices (Zhang and Wu, 2022; Zhao, 2024), with higher efficacy in collaboration but notably lower efficacy in inclusive instruction (Chen et al., 2020; Wang et al., 2018). Building on these findings, scholars have further examined the factors that shape PTs' efficacy. Among these, contact experiences with individuals with disabilities have been consistently identified as a key predictor (Wray et al., 2022). Studies indicate that PTs who have had direct contact with people with disabilities or have teaching experience with students with disabilities tend to exhibit greater efficacy for inclusive practices (Ahsan et al., 2012; Sharma and Sokal, 2015). Moreover, those who have family members or close acquaintances with disabilities often show stronger empathy and more positive attitudes toward inclusion (Mize and Schramm-Possinger, 2021; Sharma et al., 2015).

However, existing research has largely overlooked the contextual distinctions among different types of contact experience. In reality, everyday contact—such as familial or interpersonal interactions grounded in emotional closeness—may foster stable and generalized empathy and more stable affective dispositions, whereas with classmates with disabilities during schooling in school settings involves equal-status interaction within educational contexts and may therefore exert a more direct influence on beliefs related to teaching practice (Zhao et al., 2020). The lack of differentiation among contact types constrains current understanding of how distinct experiential contexts relate to PTs' efficacy beliefs. Beyond this issue of typological distinction, prior research has also given limited attention to how disability-related contact experiences may be associated with changes in efficacy over time. Much of the existing evidence is based on cross-sectional designs or variable-centered analyses that capture static associations or mean-level differences, but are less suited to examining developmental trajectories during professional preparation. As a result, it remains unclear whether and how different forms of prior contact experience are related not only to initial levels of efficacy, but also to patterns of efficacy development over time. Addressing this gap may clarify the developmental role of prior experiences and provide a more nuanced understanding of the formation of efficacy in inclusive teacher education.

Structured coursework in special or inclusive education constitutes another critical factor influencing PTs' efficacy for inclusive practices (Peebles and Mendaglio, 2014). Coursework that integrates theoretical foundations with practice-based learning—such as case analyses, simulations, and guided practicum—plays a pivotal role in developing PTs' competencies in curriculum adaptation, instructional differentiation, communication, and collaboration (Tumkaya and Miller, 2020). By contrast, coursework delivered primarily through short-term, lecture-based formats with limited opportunities for contextualized practice tends to exert only modest effects on the development of efficacy (Woodcock et al., 2012). Thus, although inclusive education coursework is widely viewed as a key mechanism for strengthening SPTs' efficacy, important questions remain regarding how instructional design and pedagogical approaches can be optimized to maximize its impact (Zhao and Peng, 2021).

Despite growing attention to the effects of coursework, the existing evidence base remains constrained by methodological limitations. Most studies rely on variable-centered approaches, typically mean comparisons, which capture overall changes in PTs' average efficacy levels but overlook heterogeneity within the population (Miesera et al., 2021). As a result, such approaches cannot identify distinct efficacy profiles or reveal how individuals with different initial profiles may follow divergent developmental trajectories over time (Xin et al., 2025). Person-centered approaches address this limitation by shifting the focus from average change to patterns of stability and transition across subgroups, enabling the examination of heterogeneous developmental pathways even when mean-level gains are observed. Importantly, these two perspectives are complementary rather than contradictory, as overall improvements may coexist with individual stability or downward transitions. Moreover, most existing studies are cross-sectional or rely on single time points, with relatively few adopting experimental or quasi-experimental designs capable of examining change associated with coursework exposure over time (Zhang and Wu, 2022; Zhao, 2024). To address these gaps, the present study employs a single-group pre–post quasi-experimental design and integrates variable-centered and person-centered analyses to examine both mean-level changes in SPTs' efficacy for inclusive practices and transitions across latent efficacy profiles over time, thereby offering a more nuanced account of how efficacy beliefs develop.

### Case-based instruction within inclusive education coursework

Case-based instruction—widely regarded as an instructional approach that meaningfully bridges theory and practice—has been shown to strengthen teachers' professional competence and teaching efficacy (Chen, 2024; Sartania et al., 2022). Rooted in authentic or simulated classroom scenarios, this approach engages PTs in case analysis, collaborative discussion, and reflective synthesis (Columbia Center for Teaching and Learning, 2019). Such structured learning processes facilitate the internalization of theoretical concepts, the development of practice-oriented instructional skills, and the cultivation of informed professional judgment in complex educational contexts (Pedagogy in Action, 2019). Although empirical research examining case-based instruction specifically within inclusive education remains limited, the pedagogical mechanisms of this approach align closely with the demands of preparing teachers for inclusive practice. Compared with traditional lecture-based coursework, case-based instruction offers several distinctive advantages for fostering PTs' efficacy for inclusive practices. First, by exposing learners to representative instructional scenarios, case-based activities provide opportunities for low-risk vicarious experiences that enable PTs to test, adapt, and apply theoretical knowledge to practical decision-making, thereby strengthening their confidence in navigating real classrooms (Nodine et al., 2024; Sharma and Nuttal, 2016). Second, the collaborative structures commonly embedded in case-based learning—such as group problem-solving and role-play—promote peer interaction, support knowledge co-construction, enhance teamwork skills, and reinforce emerging professional identities (Sartania et al., 2022). Third, cases that incorporate diverse learner perspectives and family–cultural contexts help PTs develop more profound empathy and cultivate more inclusive educational attitudes (Wu and Luo, 2020).

Existing research indicates that the effectiveness of case-based instruction depends on the coherent integration of theoretical grounding, structured reflection, and practice-based engagement (Nodine et al., 2024). Drawing on these insights, the present study embeds case-based instruction within a secondary preservice teacher education course on inclusive education. By combining the delivery of core theoretical content with representative inclusive education cases, simulation-based enactments, and guided reflective activities, this study seeks to facilitate a more holistic and sustained enhancement of SPTs' efficacy for inclusive practice.

### Present study

Building on the literature reviewed and the gaps identified above, the present study adopts a combined variable-centered and person-centered approach and integrates case-based instruction into an inclusive education coursework module. This study aims to evaluate the effectiveness of this case-based coursework in enhancing secondary SPTs' efficacy for inclusive practices, and to examine how two distinct forms of disability-related contact experiences—everyday interpersonal contact and peer contact with classmates with disabilities during schooling—shape both the magnitude of intervention effects and the developmental trajectories of efficacy profiles. Through this design, this study seeks to generate empirical evidence that can inform curriculum development and instructional innovation in inclusive secondary teacher preparation. The research questions are as follows.

1. Does a case-based inclusive education course significantly improve SPTs' efficacy for inclusive practices?
2. Do disability-related contact experiences moderate the effects of the course intervention?
3. What latent efficacy profiles emerge before and after the course, and how do these profiles change over time?
4. How do disability-related contact experiences predict transitions between latent efficacy profiles across the intervention period?

## Methods

### Participants and procedures

This study was conducted as part of a larger mixed-method project on preservice special and general education teachers' preparedness for inclusive education at a normal university in eastern China (Xin et al., 2020, 2025). Ethical approval was obtained from the University Committee on Human Research Protection at the authors' institution. The target participants in this study were SPTs enrolled in an elective inclusive education course. Participants were eligible if they (a) intended to pursue teaching in secondary schools, (b) were enrolled in teacher education programs across various subject areas (e.g., Chinese Language Arts, Mathematics, Computer Science, Chemistry), (c) had not previously taken any special or inclusive education courses, and (d) voluntarily agreed to participate. Data were collected through questionnaires administered at two time points: before the course began and immediately after its completion. Prior to survey administration, researchers informed participants that (a) all data would be used solely for academic purposes and kept confidential, (b) questionnaire responses would not affect course grades, and (c) participation was entirely voluntary, and withdrawal was permitted at any time.

A total of 551 questionnaires were distributed at the pretest, with 538 valid responses returned (97.64%). At posttest, 551 questionnaires were again distributed, yielding 524 valid responses (95.10%). To ensure data integrity, cases were retained only if participants (a) completed both pre- and posttest surveys, (b) had no systematic missing data, and (c) showed no patterned responding. The final matched sample consisted of 483 participants (87.66%). Among the participants, 335 were female (69.36%), and 148 were male (30.64%). A total of 224 participants (46.38%) reported no everyday interpersonal contact with individuals with disabilities, whereas 259 (53.62%) reported such contact (e.g., having a family member or close friend with a disability). Additionally, 316 participants (65.42%) indicated that they had not had classmates with disabilities during their schooling, while 167 (34.58%) had such classmates. Finally, 328 participants (67.91%) reported no prior knowledge of special or inclusive education policies, whereas 155 (32.10%) reported having some knowledge of these policies.

### Instruments

The Teacher Efficacy for Inclusive Practices Scale, developed by Sharma et al. (2012), was used to assess SPTs' efficacy for inclusive practices. The scale is widely employed internationally and has demonstrated strong applicability in studies involving both preservice and in-service teachers in China (Xin et al., 2025, 2026). It comprises 18 items across three dimensions—efficacy in inclusive instruction, efficacy in managing behavior, and efficacy in collaboration—with six items per dimension. Items are rated on a 6-point Likert scale (1 = strongly disagree, 6 = strongly agree), with higher scores indicating greater efficacy for inclusive practices. To evaluate the effects of the intervention, the TEIP Scale was administered at both pretest and posttest. The scale demonstrated good reliability and validity in the present study. At pretest, confirmatory factor analysis indicated acceptable model fit: χ^2^/*df* = 4.23, RMSEA = 0.08, NFI = 0.96, RFI = 0.95, IFI = 0.97, TLI = 0.96, CFI = 0.97. Cronbach's α was 0.91 for the total scale and 0.80, 0.85, and 0.85 for the three subscales. At posttest, model fit indices were χ^2^/*df* = 4.61, RMSEA = 0.08, NFI = 0.96, RFI = 0.96, IFI = 0.97, TLI = 0.96, CFI = 0.97. Cronbach's α was 0.94 for the total scale and 0.86, 0.88, and 0.87 for the three subscales.

A self-developed demographic questionnaire was administered only during the pretest. It collected information on gender, everyday interpersonal contact with individuals with disabilities, peer contact with classmates with disabilities during schooling, and other background variables (Wray et al., 2022). Following prior research, disability-related contact experiences were assessed using dichotomous (yes/no) items to indicate whether participants had engaged in everyday interpersonal contact with individuals with disabilities or had experienced peer contact with classmates with disabilities during their schooling years (Specht et al., 2016). Consistent with studies that use broad categorizations of special educational needs (Specht et al., 2016), the questionnaire did not differentiate among specific disability types. To reduce potential ambiguity in participants' interpretations, the questionnaire instructions provided a brief conceptual explanation of disability grounded in the Chinese educational context (Xin et al., 2025; Wang et al., 2018). Disability was defined as referring to individuals with psychological, physiological, or bodily impairments that result in partial or complete limitations in typical functioning, including students with visual, hearing, speech, physical, intellectual, mental, multiple, or other disabilities (General Office of The State Council of the People's Republic of China, 2021). To ensure accurate matching of pre- and posttest responses, both surveys included a student identification number as an anonymous linking code, and all personally identifying information was deleted immediately after the matching procedure.

### Case-based inclusive education coursework intervention

The case-based inclusive education coursework implemented in this study comprised 16 sessions and encompassed five core modules (see Table 1). The course was delivered by a teaching team with doctoral-level training in special education and substantial experience in inclusive education research and practice. Instruction was organized in natural class groups of no more than 40 SPTs, and case-based instruction served as the primary pedagogical approach. Each module integrated case analysis and thematic discussion grounded in systematic theoretical input. Approximately 60% of class time focused on core concepts and theoretical frameworks, with the remaining 40% devoted to case analysis and small-group discussions. During case-based activities, students were assigned to heterogeneous groups of 4–6 members, balanced by disciplinary background and prior experience. Groups adopted a rotating leadership and role-assignment system to ensure equitable participation in tasks such as note-taking, reporting, and time management. Detailed module objectives and corresponding instructional cases are presented in Table 1.

**Table 1:** Instructional design of the case-based inclusive education coursework.

**Module (number of sessions)**	**Instructional objectives**	**Sample case**
Overview of inclusive education (3 sessions)	Develop knowledge of the core concepts of inclusive education; understand inclusive perspectives on students, learning, curriculum, and instruction; examine the strengths and limitations of inclusive education; and understand the rationale and feasibility of placement in regular classrooms	I am not afraid of falling down (a case of a girl with cerebral palsy)
Student diversity and educational needs (5 sessions)	Understand the characteristics and educational needs of students with intellectual disabilities, autism spectrum disorder, attention-deficit/hyperactivity disorder, and other disabilities; and develop accurate conceptions of differences in development and learning needs between students with disabilities and their typically developing peers	The overactive Zhengwei (a case of a boy with attention-deficit/hyperactivity disorder)
Classroom management in inclusive classrooms (3 sessions)	Understand the meaning, core components, and basic strategies of classroom management in inclusive settings; recognize common challenging behaviors among students with disabilities and the underlying causes; and become familiar with basic behavior-support strategies	A boy who has difficulty staying still during music class (a case of a boy with attention-deficit/hyperactivity disorder)
Curriculum and instructional adaptation for inclusion (3 sessions)	Understand the need for curriculum and instructional adaptations; learn strategies for adapting curriculum goals, content, and organization; and understand approaches to instructional modification in inclusive classrooms	Rourou's physical education class (a case of a girl with cerebral palsy)
Communication and collaboration for inclusion (2 sessions)	Understand the importance of communication and collaboration among stakeholders in inclusive education; identify issues commonly raised by parents of students with disabilities and typically developing students; and learn strategies for effective communication and partnership	Why did parents of typically developing students jointly request the withdrawal of a child with autism?

Prior to course implementation, all instructional cases were reviewed by the teaching team to ensure that they met two essential criteria. First, each case was semi-open—originating from real classroom situations, presenting a typical instructional challenge, and allowing for more than one reasonable way to interpret or respond to the situation. Second, the cases were intervention-focused, meaning that they described an educational response that had already been implemented. SPTs were required to examine the effectiveness of this response using relevant theoretical frameworks and to propose justified improvements.

The instructional design followed a progressive sequence of theoretical grounding, practice application, conceptual consolidation, and reflective internalization, supporting SPTs' movement from knowledge acquisition to practice-oriented competence. This design was intentionally aligned with the four sources of self-efficacy proposed by Bandura (1977, 1997) in the context of inclusive teacher preparation. During the theoretical grounding phase, conceptual scaffolding strengthened mastery experiences by clarifying core concepts, legal mandates, and pedagogical principles of inclusive practices, thereby reducing ambiguity and grounding SPTs' instructional judgments. For example, in the representative case “A Boy Who Has Difficulty Staying Still During Music Class,” this phase introduced the principles and procedures of Functional Behavioral Assessment, establishing an analytic framework for understanding challenging behaviors among students with disabilities.

During the practice application phase, the course did not place SPTs in live inclusive classrooms. Instead, it drew on authentic inclusive education cases and prototypical classroom scenarios, using a structured instructional design to guide analysis and enactment. The primary aim of this phase was to provide SPTs with vicarious experiences, allowing them to explore instructional challenges in inclusive classrooms and rehearse response strategies without real-world instructional consequences. To present their analyses and proposed intervention plans, each group completed at least one case-related enactment, either as a short group role-play video or a live in-class role-play presentation. These enactments illustrated the focal classroom scenario, the strategies implemented in the original case, the challenges encountered, and the group's proposed refinements. Such simulation-based enactments were conducted as role-play activities within the university classroom and did not involve direct interaction with students with disabilities.

The conceptual consolidation phase emphasized collaborative inquiry as a form of social persuasion, with heterogeneous group discussions, presentations, peer feedback, and instructor synthesis offering professional validation, constructive challenge, and shared problem-solving focused on inclusive pedagogies. Finally, the reflective internalization phase supported affective and physiological regulation through structured reflection, as students completed guided reflection logs to integrate theoretical knowledge with practice-based insights, evaluate their responses to inclusive teaching challenges, and consolidate these experiences into more coherent and stable efficacy judgments.

### Data analysis

At the variable-centered analysis stage, paired-samples *t* tests were conducted in SPSS 27.0 to compare pretest and posttest scores on the three subscales and the total score of the TEIP Scale, thereby providing an initial assessment of mean-level changes in SPTs' efficacy for inclusive practices over time. To further examine whether these average changes varied by disability-related contact experiences, a 2 (Time: Pre- vs. post-intervention; within-subjects) × 2 (Everyday interpersonal contact with individuals with disabilities: no vs. yes; between-subjects) × 2 (Peer contact with classmates with disabilities: no vs. yes; between-subjects) mixed-design ANOVA was performed.

Whereas, variable-centered analyses focus on overall mean-level trends across the sample, at the person-centered analysis stage, latent profile analysis was conducted using Mplus 8.0 to identify the optimal number of efficacy profiles at each time point based on the three TEIP subscale scores (Masyn, 2013). Model selection was guided by multiple fit indices, including AIC, BIC, aBIC, entropy, the Lo–Mendell–Rubin likelihood ratio test (LMRT), and the bootstrap likelihood ratio test (BLRT). Lower AIC, BIC, and aBIC values indicate better model fit. Significant LMRT and BLRT values (*p* < 0.05) suggest that a *k*-class model provides a significantly better fit than a *k*−1–class model (Lo et al., 2001). Among these indices, BLRT is more robust and statistically powerful than alternative likelihood-ratio tests in mixture modeling, particularly for moderate sample sizes (Nylund et al., 2007). Entropy values closer to 1.0 indicate more precise classification. Although there are no universally accepted cut-off criteria for entropy, previous methodological literature has suggested that values above 0.80 are often regarded as indicative of good classification quality, whereas values around 0.60 are considered a commonly cited lower bound (Bauer, 2022). In addition to statistical adequacy, theoretical interpretability and sufficient class sizes were also considered in determining the final class solution (Masyn, 2013).

After establishing the optimal profile structure at both time points, latent transition analysis was employed to examine individual-level transitions between efficacy profiles, thereby capturing heterogeneity in developmental trajectories that may underlie similar mean-level trends (Collins and Lanza, 2010). Transition probability plots were used to depict patterns of movement between latent profiles, thereby capturing the developmental shifts in SPTs' efficacy for inclusive practices over time.

Finally, multinomial logistic regression analyses were conducted to assess whether everyday interpersonal contact and peer contact predicted transitions among latent profiles. Odds ratios (*OR*s) were computed to determine the likelihood of transitioning from the reference state to alternative profiles. *OR* values greater than 1 indicate an increased probability of transitioning under a given type of contact experience, whereas *OR* values below 1 indicate a decreased probability. This analysis was intended to clarify how disability-related contact experiences are differentially associated with profile-level transitions, rather than with overall mean-level change.

## Results

### Variable-centered results

As shown in Table 2, paired-sample *t* tests revealed that SPTs demonstrated statistically significant gains across all three dimensions of the TEIP Scale and the total scale score following the course implementation (*p* < 0.001). These results indicate significant increases in SPTs' efficacy for inclusive practices over time, with larger mean gains observed in overall efficacy and inclusive instruction.

**Table 2:** Pre- and post-intervention changes in SPTs' efficacy across dimensions and the total scale (*M* ± *SD*).

**Dimensions**	**Pre-intervention**	**Post-intervention**	***t*_(482)_**	**Cohens'*d***
Efficacy in inclusive instruction	4.23 ± 0.68	4.81 ± 0.63	−19.33^***^	0.88
Efficacy in managing behavior	3.94 ± 0.76	4.43 ± 0.76	−14.57^***^	0.66
Efficacy in collaboration	4.21 ± 0.80	4.81 ± 0.70	−16.40^***^	0.75
The total scale	4.13 ± 0.64	4.68 ± 0.62	−20.28^***^	0.92

^***^p < 0.001. Same for all tables.

A mixed-design ANOVA on the total efficacy score indicated a highly significant main effect of Time, *F*_(1, 479)_ = 311.17, *p* < 0.001, $\eta_{p}^{2}$ = 0.39. SPTs reported substantially higher efficacy after the intervention (*M* = 4.68, *SD* = 0.62) compared to pre-intervention levels (*M* = 4.13, *SD* = 0.64). In contrast, neither the Time × Everyday Interpersonal Contact interaction, *F*_(1, 479)_ = 0.07, *p* = 0.80, nor the Time × Peer Contact interaction, *F*_(1, 479)_ = 0.84, *p* = 0.36, was significant. The three-way interaction was also non-significant, *F*_(1, 479)_ = 1.05, *p* = 0.31. Likewise, the between-subjects main effects of everyday interpersonal contact, *F*_(1, 479)_ = 0.04, *p* = 0.85, and peer contact, *F*_(1, 479)_ = 0.08, *p* = 0.77, were not significant. These findings indicate mean-level significant improvements in SPTs' efficacy for inclusive practices over time, and these improvements did not differ across prior everyday or peer contact experiences with individuals with disabilities. The same pattern of results was replicated across all three efficacy subscales (see Table 3).

**Table 3:** Mixed-design ANOVA examining the effects of time and disability-related contact experiences on efficacy for inclusive practices (*M* ± *SD*).

**Dimensions**		**Everyday interpersonal contact**		**Peer contact**		**Time**		**Everyday interpersonal contact**		**Peer contact**		**Time** × **everyday interpersonal contact**		**Time** × **peer contact**		**Time** × **everyday interpersonal contact** × **Peer contact**	
		**No**	**Yes**	**No**	**Yes**	*F*_(1, 479)_	$\eta_{p}^{2}$	***F***_(1, 479)_	$\eta_{p}^{2}$	*F*_(1, 479)_	$\eta_{p}^{2}$	*F*_(1, 479)_	$\eta_{p}^{2}$	*F*_(1, 479)_	$\eta_{p}^{2}$	*F*_(1, 479)_	$\eta_{p}^{2}$
Efficacy in inclusive instruction	Pre-intervention	4.24 ± 0.68	4.22 ± 0.68	4.22 ± 0.66	4.24 ± 0.72	271.14^***^	0.36	0.56	0.001	0.28	0.001	0.001	< 0.001	0.05	< 0.001	0.93	0.002
	Post-intervention	4.83 ± 0.65	4.79 ± 0.61	4.80 ± 0.65	4.82 ± 0.59												
Efficacy in managing behavior	Pre-intervention	3.97 ± 0.75	3.92 ± 0.78	3.99 ± 0.74	3.84 ± 0.79	163.08^***^	0.25	0.56	0.001	0.75	0.002	0.07	< 0.001	1.97	0.004	1.94	0.004
	Post-intervention	4.45 ± 0.74	4.42 ± 0.77	4.44 ± 0.76	4.42 ± 0.75												
Efficacy in collaboration	Pre-intervention	4.20 ± 0.78	4.22 ± 0.81	4.22 ± 0.80	4.19 ± 0.80	209.12^***^	0.30	0.87	0.002	0.10	< 0.001	0.10	< 0.001	0.94	0.002	0.05	< 0.001
	Post-intervention	4.78 ± 0.71	4.84 ± 0.70	4.79 ± 0.73	4.85 ± 0.65												
The total scale	Pre-intervention	4.14 ± 0.63	4.12 ± 0.64	4.15 ± 0.63	4.09 ± 0.65	311.17^***^	0.39	0.04	< 0.001	0.08	< 0.001	0.07	< 0.001	0.84	0.002	1.05	0.002
	Post-intervention	4.69 ± 0.63	4.68 ± 0.61	4.68 ± 0.64	4.69 ± 0.58												

^***^p < 0.001.

### Person-centered results

Latent profile analyses were conducted to identify the optimal class structure for SPT's efficacy for inclusive practices at pre- and post-intervention. At both time points, the model enumeration explicitly considered the three- and four-class solutions as competing alternatives, in addition to the two-class baseline, to identify the most appropriate representation of heterogeneity in SPTs' efficacy profiles (see Table 4).

**Table 4:** Model fit statistics for the pre- and post-intervention latent profile analyses.

**Time**	**Model**	**AIC**	**BIC**	**aBIC**	**Entropy**	**LMRT(*p*)**	**BLRT(*p*)**	**Profile membership probabilities**
Pre-intervention	1	3,267.86	3,292.94	3,273.89	–	–	–	1
	2	2,937.86	2,979.66	2,947.92	0.72	< 0.001	< 0.001	0.60/0.40
	3	2,852.35	2,910.87	2,866.44	0.71	0.19	< 0.001	0.18/0.26/0.56
	4	2,810.40	2,885.64	2,828.51	0.77	0.51	< 0.001	0.50/0.36/0.08/0.07
Post-intervention	1	3,061.49	3,086.57	3,067.52	–	–	–	1
	2	2,617.96	2,659.76	2,628.02	0.76	< 0.001	< 0.001	0.43/0.57
	3	2,451.38	2,509.90	2,465.47	0.79	0.02	< 0.001	0.24/0.20/0.56
	4	2,378.04	2,453.28	2,396.15	0.84	0.20	< 0.001	0.31/0.03/0.16/0.50

At pre-intervention, the three-class model showed lower AIC, BIC, and aBIC values than the two-class model, indicating a better fit. Although the four-class model yielded marginally lower information criteria and higher entropy (0.77 vs. 0.71), it produced two small classes, each representing approximately 7% and 8% of the sample, respectively. Such small class sizes raise concerns about potential overextraction and limited analytic and substantive interpretability. Model comparison tests further supported the three-class solution. Specifically, the BLRT indicated a significant improvement of the three-class model over the two-class model (*p* < 0.001). In contrast, according to the LMRT, adding a fourth class did not result in a statistically significant improvement in model fit (*p* = 0.51). Beyond statistical fit indices, the three-class solution produced profiles that were theoretically coherent and readily interpretable as low-, moderate-, and high-efficacy groups, consistent with prior research and conceptual expectations (Xin et al., 2025). Taken together, these findings suggest that, despite marginal statistical advantages of the four-class model on selected indices, the three-class model provided a more parsimonious and substantively meaningful representation of pre-intervention efficacy profiles.

A comparable pattern emerged at post-intervention. Both the three- and four-class models showed markedly improved fit relative to the two-class model. The three-class solution was supported by both the BLRT (*p* < 0.001) and the LMRT (*p* = 0.02), and demonstrated good classification quality (entropy = 0.79) with reasonably balanced class proportions. Although the four-class model again exhibited slightly lower AIC values and higher entropy (0.84), it included one very small class representing approximately 3% of the sample and did not yield a statistically significant improvement over the three-class solution according to the LMRT (*p* = 0.20). The presence of such a very small class further suggests potential overfitting and limited utility for substantive interpretation or longitudinal analysis. Importantly, the three-class solution at post-intervention retained the same theoretically expected low-, moderate-, and high-efficacy profiles identified at pre-intervention, thereby facilitating meaningful longitudinal comparability across time points. By contrast, the additional class introduced in the four-class solution did not provide clear conceptual differentiation beyond subdividing existing profiles.

**Figure 1:**
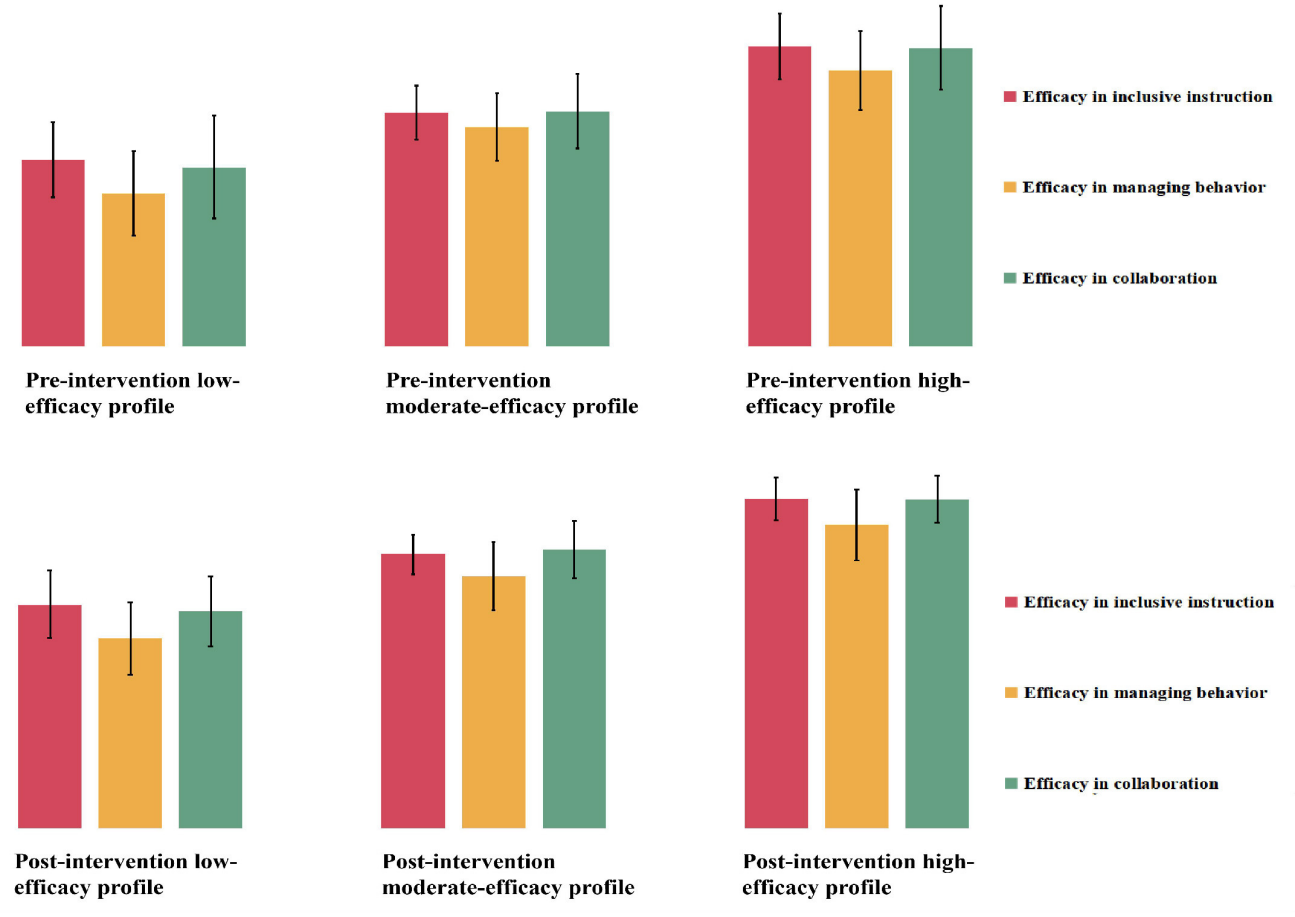
Latent profile shapes for the selected three-class solution of SPTs' efficacy for inclusive practices at pre- and post-intervention.

Accordingly, the three-class model was selected as the optimal solution at both pre- and post-intervention, reflecting a balanced consideration of statistical fit, classification quality, parsimony, and theoretical interpretability. Although the four-class model was carefully evaluated as a competing alternative, it was not retained due to very small classes, insufficient statistical support for additional complexity, and limited incremental conceptual value. Figure 1 illustrates the latent profile shapes of the selected three-class solution by plotting mean scores on the three efficacy dimensions for each profile, thereby facilitating interpretation of the substantive meaning of each latent group.

**Figure 2:**
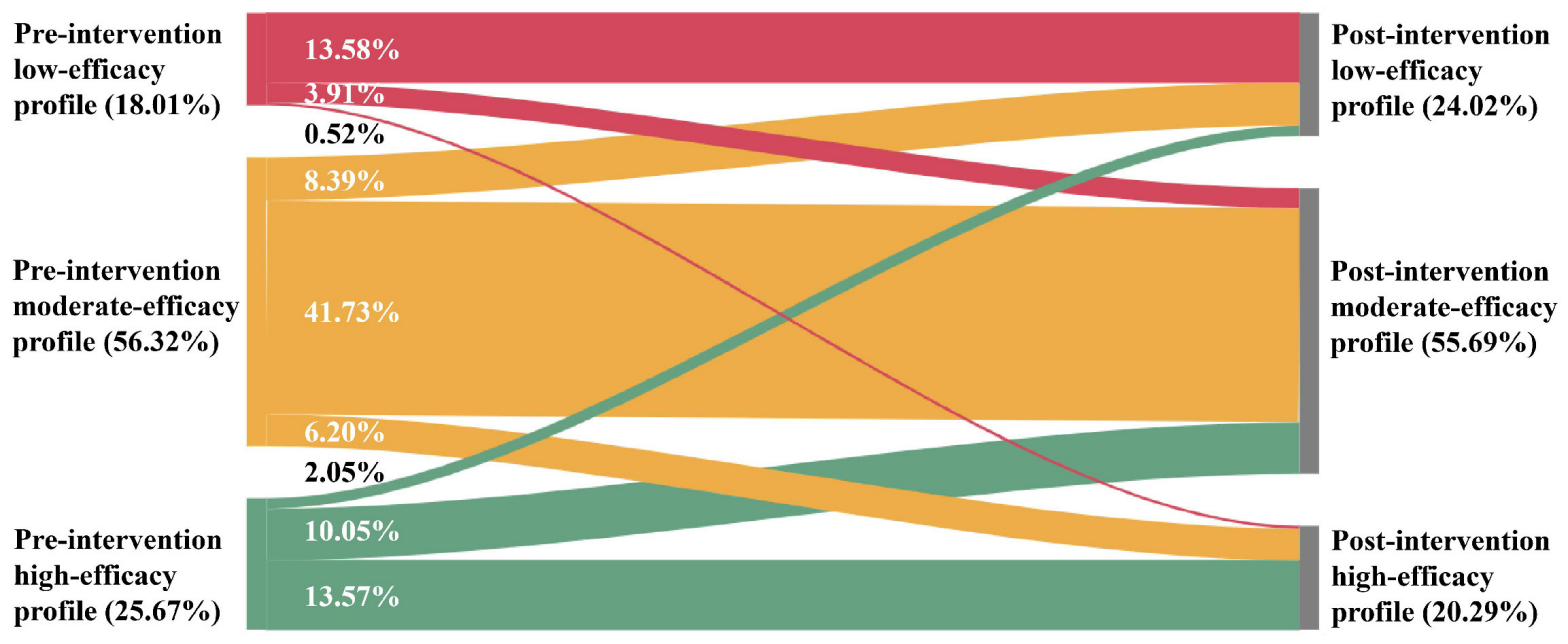
Latent profile probabilities and transition probabilities from pre- to post-intervention.

Figure 2 depicts the latent transition results for the three efficacy profiles across the intervention period. Overall, profile membership exhibited both stability and observable movement over time. The pre-intervention low-efficacy profile (18.01% of the sample) was the most stable, with 15.58% of participants remaining in the low-efficacy profile at posttest, while smaller proportions transitioned to the moderate-efficacy profile (3.91%) or the high-efficacy profile (0.52%). At a descriptive level, profile-level mean scores associated with the low-efficacy profile were higher at posttest than at pre-intervention (see Figure 1), reflecting differences in the score distributions associated with this profile across time points rather than formally tested within-profile change. The pre-intervention moderate-efficacy profile (56.32% of the sample) also showed substantial stability. A majority of participants (41.73%) remained in the same profile, whereas 8.39% transitioned to the low-efficacy profile and 6.20% transitioned to the high-efficacy profile. Descriptively, participants transitioning into higher-efficacy profiles were associated with higher profile-level mean scores at posttest, whereas those transitioning into lower-efficacy profiles were associated with lower profile-level mean scores (see Figure 1), indicating systematic differences in profile-level score distributions across transition patterns rather than inferential evidence of within-profile change over time. The pre-intervention high-efficacy profile (25.67% of the sample) was comparatively less stable. At posttest, 13.57% of participants retained membership in the high-efficacy profile, while 10.05% transitioned to the moderate-efficacy profile and 2.05% transitioned to the low-efficacy profile. Across these transition groups, descriptive differences in profile-level mean score distributions were observed at posttest relative to pre-intervention (see Figure 1), suggesting heterogeneity in how efficacy profiles were reorganized over the intervention period. Taken together and alongside the overall mean-level increases observed in the variable-centered analyses, the latent transition results suggest that efficacy development during the intervention period was accompanied by changes in profile membership and distribution, rather than uniform developmental trajectories across individuals.

Multinomial logistic regression analyses were conducted to examine whether baseline everyday interpersonal contact and peer contact with classmates with disabilities predicted transitions among latent efficacy profiles (see Table 5). Both predictors were dummy-coded (0 = no contact, 1 = contact), and transition outcomes were coded with “no change in profile membership” (e.g., pre-low → post-low) as the reference category. With the pre-low → post-low transition as the reference, peer contact significantly reduced the odds of transitioning from the low to the moderate profile (*B* = −1.11, *SE* = 0.52, *p* = 0.03; *OR* = 0.33, 95% CI [0.12, 0.89]), indicating that SPTts who had peer contact during schooling were less likely than those without such contact to move from low to moderate efficacy after the course. Baseline everyday interpersonal contact did not significantly predict transitions from the low profile to either the moderate or high profiles (*ps* > 0.05). Using the pre-moderate → post-moderate transition as the reference, baseline everyday interpersonal contact was associated with increased odds of both downward and upward movement. SPTs with everyday interpersonal contact had higher odds of moving from moderate to low (*B* = 0.97, *SE* = 0.35, *p* = 0.006; *OR* = 2.64, 95% CI [1.32, 5.29]) and of moving from moderate to high (*B* = 1.41, *SE* = 0.56, *p* = 0.01; *OR* = 4.10, 95% CI [1.36, 12.29]). Peer contact did not significantly predict transitions out of the moderate profile (*ps* > 0.05). Finally, with the pre-high → post-high transition as the reference, neither baseline everyday interpersonal contact nor peer contact significantly predicted transitions from the high profile to either the moderate or low profiles (*ps* > 0.05).

**Table 5:** Multinomial logistic regression of the effects of two types of contact experiences on latent profile transitions.

**Profiles**	**Disability-related contact experiences**	**Post-intervention low-efficacy profile**			**Post-intervention moderate-efficacy profile**			**Post-intervention high-efficacy profile**		
		***B*** **(*****SE*****)**	***OR***	**95% CI**	***B*** **(*****SE*****)**	***OR***	**95% CI**	***B*** **(*****SE*****)**	***OR***	**95% CI**
Pre-intervention low-efficacy profile	Everyday interpersonal contact	–	–	–	−0.66 (0.54)	0.52	[0.18, 1.51]	0.16 (1.45)	1.18	[0.07, 20.36]
	Peer contact	–	–	–	−1.11^*^ (0.52)	0.33	[0.12, 0.91]	−0.56 (1.45)	0.57	[0.03, 9.90]
Pre-intervention moderate-efficacy profile	Everyday interpersonal contact	0.97^**^ (0.35)	2.64	[1.34, 5.21]	–	–	–	1.41^*^ (0.56)	4.10	[1.36, 12.39]
	Peer contact	0.003 (0.39)	1.00	[0.47, 2.13]	–	–	–	−0.94 (0.56)	0.39	[0.13, 1.17]
Pre-intervention high-efficacy profile	Everyday interpersonal contact	−0.15 (0.76)	0.86	[0.19, 3.83]	0.12 (0.38)	1.12	[0.53, 2.39]	–	–	–
	Peer contact	1.00 (1.12)	2.72	[0.30, 24.44]	−0.54 (0.41)	0.58	[0.26, 1.30]	–	–	–

^*^p < 0.05, ^**^p < 0.01. Some estimated odds ratios are associated with wide confidence intervals, likely due to small sample sizes in specific profile transition cells (e.g., low-to-high or high-to-low transitions). These estimates may be unstable and should be interpreted with caution.

## Discussion

The present study advances understanding of how SPTs develop efficacy for inclusive practices by integrating variable-centered and person-centered analyses within a case-based inclusive education coursework intervention. The variable-centered findings indicated significant overall gains in efficacy over time, reinforcing international evidence that structured, practice-oriented coursework is associated with stronger inclusive teaching competence. At the same time, the identification of three distinct efficacy profiles and their partially dynamic transitions shows that these gains do not unfold uniformly, adding a more differentiated, person-centered perspective to existing research. Disability-related interpersonal and peer contact were differentially associated with profile transitions, suggesting that these contacts may serve as contextual factors shaping how SPTs recalibrate their efficacy during learning. These findings underscore the value of complementing mean-level analyses with person-centered approaches to capture heterogeneous developmental pathways and to inform more responsive designs for inclusive teacher preparation.

### Overall efficacy gains following case-based coursework

The present study provides variable-centered evidence of significant increases in SPTs' efficacy following participation in a case-based inclusive education course, offering new empirical support from the Chinese context and reinforcing the widely recognized importance of coursework in preparing teachers for inclusive practice (Wray et al., 2022). After completing a 16-session case-based curriculum, SPTs showed significant gains in their efficacy for inclusive practices. This finding aligns with Swain et al. (2012), who emphasized that coursework integrating theoretical and practical components can meaningfully promote PTs' efficacy for inclusive practices. It also speaks to concerns raised by Zhao and Peng (2021), who argued that theory-only courses have a limited impact on PTs' efficacy for inclusive practices. Taken together, the observed gains underscore the potential value of case-based pedagogies for bridging theoretical knowledge and the practical demands of inclusive teaching (Nodine et al., 2024). In this context, as described in the intervention section, the coursework was intentionally structured around conceptual scaffolding, case-based application, collaborative inquiry, and structured reflection, providing a plausible instructional basis for the observed mean-level gains in SPTs' efficacy for inclusive practices.

A notable variable-centered finding is that neither the interaction between time and everyday interpersonal contact nor between time and peer contact with individuals with disabilities was significant, and the three-way interaction was also non-significant. This pattern indicates that observed efficacy gains over time were not differentially associated with SPTs' prior disability-related contact experiences. Accordingly, the case-based curriculum appears to be associated with comparable patterns of improvement for individuals with and without such experiences. One possible interpretation is that structured, case-based coursework may help transform diverse personal experiences into more coherent professional understandings, providing an accessible pathway for professional growth across heterogeneous groups of SPTs (Peebles and Mendaglio, 2014).

The course was intentionally designed around four interrelated components—conceptual scaffolding, case-based application, collaborative inquiry, and structured reflection—which together operationalize and extend the four sources of self-efficacy proposed by Bandura (1977, 1997) within the context of inclusive teacher preparation. Conceptual scaffolding strengthened mastery experiences by clarifying core concepts, legal mandates, and pedagogical principles of inclusive practices, thereby reducing ambiguity and grounding SPTs' instructional judgments. Case-based application generated vicarious experiences by situating SPTs in realistic, inclusive classroom dilemmas, allowing them to observe, analyze, and mentally rehearse responsive strategies without real-world consequences. Collaborative inquiry functioned as social persuasion, as heterogeneous group discussions offered professional validation, constructive challenge, and shared problem-solving focused on inclusive pedagogies. Structured reflection supported affective and physiological regulation by guiding SPTs to integrate coursework insights, evaluate their responses to inclusive teaching challenges, and consolidate these experiences into coherent and stable efficacy judgments. These design elements demonstrate how a case-based inclusive education curriculum can enact and extend the core assumptions of Bandura's (1977, 1997) social cognitive theory by simultaneously activating all four sources of self-efficacy within a coherent instructional system.

### Developmental stability and cognitive recalibration in efficacy profile transitions

Person-centered analyses revealed a more differentiated developmental pattern than variable-centered gains alone imply, offering methodological insight into how such analyses capture heterogeneity and developmental asymmetries that conventional mean-level methods tend to obscure (Swain et al., 2012; Zhao and Peng, 2021). Descriptively, efficacy levels were generally higher at post-intervention than at pre-intervention across all profiles, suggesting overall improvement rather than uniform stagnation. Nevertheless, a substantial proportion of SPTs in the low- and moderate-efficacy profiles remained in their initial categories over the intervention period, indicating a pattern of relative profile stability rather than rapid convergence across groups. This pattern aligns with social-cognitive accounts of efficacy as a belief system anchored in pre-existing schemas, accumulated mastery, and judgments of instructional competence (Bandura, 1977, 1997). Accordingly, stability of profile membership should not be interpreted as a lack of development. Short-term coursework may primarily support refinement within existing efficacy structures, whereas more pronounced reorganization across profiles is likely to require extended practicum exposure, repeated mastery experiences, and sustained feedback cycles (Swain et al., 2012). From this perspective, the limited degree of cross-profile movement observed in the present study is theoretically plausible. SPTs who began with lower efficacy had to overcome steeper learning demands, while a ceiling effect naturally limited those who started at higher levels. Within the time frame of a brief intervention, rapid convergence across profiles was therefore unlikely. Although the case-based curriculum appeared to strengthen conceptual understanding and situational reasoning, coursework alone may be insufficient to reorganize deeper assumptions about teaching capacity, the educability of students with disabilities, or one's regulatory resources in complex instructional contexts (Zhao and Peng, 2021).

Although the proportion was smaller than that observed in the low- and moderate-efficacy profiles, most SPTs in the high-efficacy profile remained in the same category across the intervention period. Consistent increases in mean efficacy scores were observed descriptively within this profile from pre- to post-intervention, suggesting upward shifts at the profile level among individuals who remained in the high-efficacy group. While these changes were not formally tested for statistical significance, they indicate that efficacy development may also occur among SPTs who entered the course with relatively high initial efficacy.

Against this backdrop of overall improvement, the downward transitions observed among a subset of high-efficacy SPTs may be interpreted as reflecting a process of cognitive recalibration rather than diminished capability. This interpretation is theoretically grounded in social cognitive theory and stage-based models of teacher development, which suggest that early efficacy beliefs—particularly in the absence of extensive teaching experience—may be optimistic or weakly calibrated (Bandura, 1977, 1997; Fuller, 1969). Exposure to realistic, discipline-specific inclusion challenges during coursework may prompt more critical self-appraisal and refinement of efficacy judgments, especially when cases involve academic adaptation, behavioral complexity, and multi-stakeholder coordination. In this sense, downward transitions are more appropriately understood as reflecting shifts in evaluative standards rather than declines in competence, although alternative explanations, such as contextual influences or measurement-related factors, cannot be ruled out. Consistent with developmental models of teacher learning, this pattern may represent a normative adjustment process, in which initial confidence gives way to more calibrated, professionally grounded judgments (Korthagen and Vasalos, 2005). It should be noted that this interpretation is inferential and based on quantitative transition patterns rather than direct qualitative evidence, and therefore represents one plausible theoretical account rather than a definitive explanation.

### Disability-related contact and differential efficacy pathways

At the profile level, person-centered analyses extend prior variable-centered work by showing that disability-related contact experiences do not uniformly foster efficacy growth—as often implied in earlier research (Ahsan et al., 2012; Sharma and Sokal, 2015)—but instead are differentially associated with developmental pathways. Specifically, peer contact significantly reduced the likelihood that SPTs in the low-efficacy profile would transition to the moderate profile. Importantly, this pattern should not be interpreted as an absence of efficacy-related change, as SPTs with prior peer contact still exhibited stability within the low-efficacy profile rather than categorical movement across profiles. From a person-centered perspective, this suggests that developmental change may take the form of within-profile refinement rather than profile transition. In this sense, peer contact may primarily serve as a stabilizing constraint on profile membership, shaping the pattern of efficacy development rather than its direction (Xin et al., 2024b). Consistent with intergroup contact theory, the direction of contact effects depends on the quality of contact (Zhao et al., 2020). In the Chinese context, earlier phases of inclusive education were marked by limited resources and inconsistent implementation (Zhang and Miao, 2022), which likely shaped the nature of peer interactions experienced by the current cohort of SPTs during their own schooling. Empirical studies from this period indicate that students with disabilities in regular schools often occupied marginal positions within classroom social networks and experienced low levels of peer acceptance (Xie, 2018). Such peer dynamics were embedded within a broader school ecology characterized by relatively low parental acceptance of inclusive placement among parents of typically developing students (Yan, 2009; Xu and Zhao, 2017) and by a tendency for general education teachers to endorse inclusion at an attitudinal level while engaging less consistently in inclusive practices at the behavioral level (Jiang, 2014). Within these conditions, peer contact may have been shaped less by supportive engagement and more by social distance or subtle exclusion, increasing the likelihood that early contact experiences contributed to the consolidation of cautious or deficit-oriented expectations toward students with disabilities (Xin et al., 2025). In such contexts, the conceptual and strategic tools provided by short-term coursework may be insufficient to offset cognitions shaped by cumulative peer contact experiences.

In contrast, disability-related interpersonal contact in everyday life was differentially associated with efficacy patterns among SPTs in the moderate-efficacy profile. On the one hand, such contact increased the likelihood of a downward transition to the low-efficacy profile; on the other hand, it also heightened the probability of a move into the high-efficacy profile. This pattern suggests that interpersonal contact carries strong emotional salience, but the direction of its impact varies depending on the nature of prior interactions, the individual's emotional regulation capacity, and existing professional schemas (Mize and Schramm-Possinger, 2021; Sharma et al., 2015). For some SPTs, emotionally charged encounters may amplify empathy for families' difficulties, prompting more cautious self-evaluation and a more conservative sense of capability. For others, interpersonal connections may strengthen a sense of mission and prosocial motivation, thereby promoting substantial growth in efficacy.

### Implications for teacher education

Inclusive teacher education programs should adopt a structured, case-based curriculum that explicitly situates theory, authentic dilemmas, collaborative inquiry, and guided reflection within the context of inclusive education. Such designs may support efficacy development by helping SPTs engage systematically and reflectively with the complexities of inclusive teaching, without relying heavily on prior disability-related experience, and may offer a potentially scalable framework for supporting SPTs' professional development.

Inclusive teacher education programs should differentiate preparation pathways according to SPTs' initial efficacy profiles. Low-efficacy SPTs may require high-structure scaffolds—such as guided case walkthroughs, micro-teaching rehearsal, and designed mastery experiences—to build baseline confidence for inclusive practice. For those with moderate efficacy, more complex cases, deeper analytic reasoning, and sustained reflective dialogue can foster conceptual advancement. For high-efficacy SPTs, exposure to more complex and ambiguous inclusion scenarios, such as interdisciplinary collaboration or multi-agency coordination, may offer opportunities for further reflection and professional growth when accompanied by appropriate expert feedback. Such tiered designs reflect developmental models of teacher learning and promote calibrated, professionally grounded efficacy for inclusive teaching.

Inclusive teacher education programs should incorporate mechanisms that address emotional and experiential aspects through which disability-related contact shapes SPTs' developmental trajectories. For low-efficacy SPTs with negative peer contact histories, coursework may need to provide high-quality case demonstrations, positive exemplars of inclusive peer interaction, and structured cognitive reframing to disrupt deficit-based beliefs. For moderate-efficacy SPTs whose interpersonal contact is emotionally salient, training in emotion recognition, regulation, and perspective-taking can prevent overload for some while channeling prosocial motivation for others. Across all profiles, case-based instruction may benefit from embedding systematic analysis of contact quality—such as emotional valence, interaction depth, and relational dynamics—to cultivate the capacity to interpret and construct positive contact in inclusive school settings.

### Limitations and future research directions

This study has several limitations. First, the use of a single-group pre–post quasi-experimental design without a comparison group limits the strength of causal inference. Although improvements in SPTs' efficacy were observed following the implementation of the case-based coursework, these changes cannot be confidently attributed solely to the intervention. Other potential influences, including concurrent coursework, additional professional learning experiences, and natural developmental changes over time, may also have contributed to the observed gains. Accordingly, the findings should be interpreted as reflecting a strong association between course participation and improvement in efficacy, rather than as definitive evidence of broad or generalized causal effects.

Second, only immediate post-intervention outcomes were assessed, leaving the durability and longer-term developmental trajectory of efficacy change unexamined. Future research should incorporate control groups within more rigorous experimental designs and employ longitudinal follow-ups that track SPTs through graduation and into the early career stage to establish more robust evidence for the sustained impact of case-based inclusive education coursework on the development of efficacy for inclusive practices.

Third, disability-related contact experiences were assessed using broad, dichotomous indicators that did not distinguish between disability types or qualitative aspects of contact. Although this approach aligns with prior research and common practice in large-scale studies (Specht et al., 2016), participants' interpretations of disability and their contact experiences may have varied, potentially obscuring differences in how contact relates to efficacy in inclusive practices. In particular, different disability types and variations in contact depth, duration, and voluntariness may be differentially associated with efficacy for inclusive practices. Future research would benefit from more fine-grained measures that capture both the type and quality of disability-related contact experiences.

Fourth, although disability-related contact experiences were examined as predictors of transitions between extracted efficacy profiles, these variables were not directly incorporated into the latent transition model. This reflects the fact that prior research has primarily focused on associations between experiences and levels of self-efficacy, with less attention to their role in transitions in self-efficacy over time (Ahsan et al., 2012; Mize and Schramm-Possinger, 2021; Sharma et al., 2015; Sharma and Sokal, 2015). As a result, the present analyses relied on assigned profile membership without fully accounting for classification uncertainty in class prevalences, which warrants caution in interpreting the results of the latent transition analysis (Nylund-Gibson et al., 2014, 2023). This limitation is further evident in the baseline entropy values, where the pre-intervention entropy (0.71), although within an acceptable range, indicates lower classification precision than the post-intervention entropy (0.79). Accordingly, the estimated transition probabilities should be interpreted with appropriate caution.

Finally, some estimated odds ratios had wide confidence intervals, notably for everyday interpersonal contact predicting transitions from the pre-intervention low-efficacy profile to the post-intervention high-efficacy profile, and for peer contact predicting transitions from the pre-intervention high-efficacy profile to the post-intervention low-efficacy profile. These wide confidence intervals likely reflect small sample sizes in the corresponding transition cells, indicating instability in parameter estimation and warranting caution in interpretation.

## Data Availability

The raw data supporting the conclusions of this article will be made available by the authors, without undue reservation.
